# Down-regulated TAB1 suppresses the replication of Coxsackievirus B5 via activating the NF-κB pathways through interaction with viral 3D polymerase

**DOI:** 10.1186/s12985-023-02259-w

**Published:** 2023-12-10

**Authors:** Jiayu Zhang, Peiying Teng, Bo Sun, Jihong Zhang, Xiaoshuang Zhou, Wei Chen

**Affiliations:** Medical School, Kunming University of Science and Technology, No. 727, Southern Jingming Road, Chenggong District, Kunming, 650500 Yunnan Province People’s Republic of China

**Keywords:** Hand-foot-mouth disease (HFMD), Coxsackievirus group B type 5 (CVB5), 3D polymerase, TGF-BATA-activated kinase1 binding protein 1 (TAB1), Nuclear factor kappa-B (NF-κB) pathway

## Abstract

Coxsackievirus Group B type 5 (CVB5), an important pathogen of hand-foot-mouth disease, is also associated with neurological complications and poses a public health threat to young infants. Among the CVB5 proteins, the nonstructural protein 3D, known as the Enteroviral RNA-dependent RNA polymerase, is mainly involved in viral genome replication and transcription. In this study, we performed immunoprecipitation coupled with liquid chromatography-tandem mass spectrometry (LC–MS/MS) to identify host proteins that interacted with CVB5 3D polymerase. A total of 116 differentially expressed proteins were obtained. Gene Ontology analysis identified that the proteins were involved in cell development and cell adhesion, distributed in the desmosome and envelope, and participated in GTPase binding. Kyoto Encyclopedia of Genes and Genomes analysis further revealed they participated in nerve diseases, such as Parkinson disease. Among them, 35 proteins were significantly differentially expressed and the cellular protein TGF-BATA-activated kinase1 binding protein 1 (TAB1) was found to be specifically interacting with the 3D polymerase. 3D polymerase facilitated the entry of TAB1 into the nucleus and down-regulated TAB1 expression via the lysosomal pathway. In addition, TAB1 inhibited CVB5 replication via inducing inflammatory factors and activated the NF-κB pathway through IκBα phosphorylation. Moreover, the 90-96aa domain of TAB1 was an important structure for the function. Collectively, our findings demonstrate the mechanism by which cellular TAB1 inhibits the CVB5 replication via activation of the host innate immune response, providing a novel insight into the virus-host innate immunity.

**Supplementary Information:**

The online version contains supplementary material available at 10.1186/s12985-023-02259-w.

## Background

Hand-foot-and-mouth disease (HFMD) commonly occurs in children younger than five years between the spring and fall and transmits by fecal–oral, and oral-oral, contact as well as via respiratory. In most cases, the common symptoms presented are self-limited febrile illness with a low-grade fever, maculopapular rash on the hands and feet, and painful oral ulcerations. HFMD has been linked to human enteroviruses 71 (EV71) and coxsackieviruses (CV, types A6, A16, B2, and B5) with EV71 and CVA16 historically the most common causes of HFMD [1]. However, there has been a recent emergence of CVB5-associated outbreaks worldwide, including in Asia, Europe, and South America [2–4]. In rare cases, CVB5-infected infants may also present with neurologic complications, such as aseptic meningitis and viral encephalitis, which lead to high mortality [5]. In northeastern Poland, CVB5 accounted for 32% of enterovirus types infected in the central nervous system of children in the last 5 years [6]. In Brazil, the CVB5 strain had been derived from cerebrospinal fluid and stool samples from patients with aseptic meningitis or acute flaccid paralysis [2]. In the analysis of viral meningitis cases in China (Yunnan province), 70% of the cases were caused by enterovirus infection, among which CVB5 accounted for the highest proportion [7]. Consequently, the development of antiviral drugs and vaccines is critical to prevent CVB5 infection.

CVB5 belongs to the genus Enterovirus of the *Picornaviridae* family and is a non-enveloped with single-stranded positive-strand RNA (+ ssRNA). The viral genome has 7400 bp and encodes a polyprotein precursor of 2194 amino acids, which is further hydrolyzed into three precursor proteins (P1, P2, P3). The P1 precursor protein encodes four structural proteins (VP1, VP2, VP3, and VP4) mainly responsible for the assembly of the viral nucleocapsid. Among them, the VP1 protein has the main neutralizing antigenic determinants and is important for enterovirus serotyping. The P2 and P3 gene regions encode seven nonstructural proteins (NSPs) (2A, 2B, 2C, 3A, 3B, 3C, and 3D). The NSPs have enzymatic activities that block receptor-recognized signaling pathways and allow the virus to escape the innate immune response [8]. Among the NSPs, 2A and 3C have been extensively studied in the *Picornavirus* family. For example, EV71 3C binding to RIG-I blocks the downstream pathways and inhibits the production of proinflammatory factors, thereby allowing the virus to escape the innate immune response [9]. And EV-D68 2A inhibits the type I interferon (IFN-I) response by cleaving tumor necrosis factor receptor-associated factor 3 (TRAF3) to promote virus survival [10]. Moreover, in other viruses, NSPs also have similar functions in regulating innate immune signaling pathways. For example, the duck Tembusu virus (DTMUV) NS3 induces autophagy and promotes viral replication through extracellular signal-regulated kinase 2 (ERK2), phosphatidylinositol-3 kinase (PI3K)/AKT and mammalian target of rapamycin (mTOR) signaling pathways [11]. Therefore, studying the role of NSPs in regulating the innate immune pathways is vital for viral replication.

The nuclear factor kappa-B (NF-κB) is an important immune response protein consisting of five family members (p65/RelA, RelB, cRel, p50, and p52). They are able to regulate cell differentiation, survival, and proliferation, as well as transcription of cytokines [12]. In the resting state, NF-κB/Rel transcription factors form complexes with IκB proteins and present in the cytoplasm in an inactive state. Cell surface-specific receptors are recognized after the virus infected, then activate the downstream adaptor proteins and NF-κB signaling pathways to resist pathogen invasion by promoting the production of inflammatory cytokines [13]. For example, EV71 2C protein suppresses IκB kinase β phosphorylation, thereby inhibiting NF-κB activation and allowing the virus to escape [14]. CVA16 2C recruits protein phosphatase 1, inhibiting IKKβ phosphorylation to regulate the NF-κB pathway and viral replication [15]. However, the regulatory mechanism of the NF-κB signaling pathway through CVB5 NSPs has not been reported.

Liquid chromatography-tandem mass spectrometry (LC–MS/MS) is an analytical chemistry technique and can be used to analyze protein sequences, sites of protein post-translational modifications, and for quantitative proteomics [16, 17]. Therefore, in this study, we used LC–MS/MS coupled with immunoprecipitation to identify the host proteins that interact with the CVB5 3D polymerase. Through the bioinformatics analysis and experimental verification, we selected the candidate protein TGF-BATA-activated kinase1 binding protein 1 (TAB1) for further study. Our findings suggested that 3D polymerase facilitated the entry of TAB1 into the nucleus and down-regulated TAB1 expression via the lysosomal pathway. Also, the TAB1 inhibited the replication of CVB5 by enhanced inflammatory factors and activated the NF-κB pathway through the IκBα phosphorylation. Our study revealed a new antiviral mechanism for cellular TAB1 protein and provided a scientific basis for the development of drugs against CVB5 infection.

## Materials and methods

### Cells and viral infection

Human rhabdomyosarcoma cells (RD) and human embryonic kidney 293 T (HEK293T) cells were cultured in Dulbecco’s modified Eagle medium (DMEM) supplemented with 10% heat-inactivated fetal bovine serum (FBS) incubated in CO_2_ incubator at 37 °C.

The CVB5 strain (GenBank accession no. MH201081.1) was isolated from the HFMD patient in Kunming, Yunnan province in 2014. To determine the viral 50% Tissue Culture Infectious Dose (TCID_50_), RD cells were inoculated and infected with 100 μL of the serially diluted virus, then cultured for 3-7d. CVB5 TCID_50_ was measured by Reed's and Muench's method.

### Plasmids and transfection

The CVB5 3D polymerase gene was cloned into the pcDNA3.1 vector carrying the Flag tag (named pcDNA3.1-3D-2Flag). TAB1, the 90-96aa domain of TAB1 and its mutant were cloned into the pcDNA3.1 plasmid (named pcDNA3.1-TAB1, pcDNA3.1-TAB1 (69–334) and pcDNA3.1- TAB1-ALA, respectively). TAB1 siRNA was synthesized as follows: si-TAB1-1: GCACTTTTATGCAAATCGA, si-TAB1-2: GCAACCGAGTGACCAACTT, and si-TAB1-3: GGATGAGCTCTTCCGTCTT (Ribobio, China).

Cells were cultured until 70–80% confluency and the 2.0 μg plasmids (or 50 nmol/L si-TAB1) were transfected with Lipofectamine 2000 (11,668, Thermo Fisher, USA), then after 24 h infected CVB5 (MOI = 0.1). Cells were harvested at 24 h post-infection (h.p.i.) and lysed. Mock-infected cells were used as the control.

### Sample preparation and protein extraction

RD cells transfected with the 2.0 μg pcDNA3.1-3D-2Flag (pcDNA3.1 as the control) and samples were harvested after 24 h (three biological replicates for each group). SDT (4%SDS, 100 mM Tris–HCl, 1 mM DTT, pH 7.6) lysis buffer was used for proteins extraction. The amount of protein was quantified using the bicinchoninic acid (BCA) protein assay kit (PA115-02, Tiangen Biothch, China). Protein samples were digested by trypsin according to the filter-aided sample preparation (FASP) procedure [18]. The digesting peptides in each sample were desalted on C18 Cartridges (Empore™ SPE Cartridges C18, Sigma, USA), concentrated by vacuum centrifugation, and reconstituted in 40 µL of 0.1% (*v*/*v*) formic acid.

### LC–MS/MS analysis

LC–MS/MS analysis was performed on a Q Exactive mass spectrometer (0726090, Thermo Scientific, USA) coupled to Easy nLC (LC140, Thermo Scientific, USA). Peptides were loaded onto a reverse-phase capture column (nanoViper C18, Thermo Scientific, USA) for linear gradient separation. The isolated peptides were analyzed in a Q Exactive mass spectrometer operating in positive ion detection mode with peptide recognition mode enabled. The experiment was performed with the assistance of Applied Protein Technology Co., Ltd, China.

### Data processing and bioinformatic analysis

The raw mass spectrometry data for each sample was combined, identified, and quantified using the MaxQuant 1.5.3.17 software (https://www.maxquant.org/). Protein sequences of selected differentially expressed proteins were searched for using the NCBI BLAST + client software (NCBI-blast-2.2.28 + -win32.exe) and InterProScan (https://www.ebi.ac.uk/interpro/) [19]. To analyze the function of differentially expressed proteins, mapping of gene ontology (GO) terms biological process (BP), cellular component (CC), and molecular function (MF) were determined using Blast2GO [20], annotated using the Kyoto Encyclopedia of Genes and Genomes (KEGG) online database (http://geneontology.org/) and mapped to pathways [21], and the protein–protein interaction (PPI) information was retrieved with STRING (http://string-db.org/) [22]. Also, based on the Fold change and p-value (T-test), Volcano maps for the significantly differentially proteins.

### Immunoprecipitation and immunoblotting

Cells were transfected with plasmids, collected, and lysed. The special antibody (TAB1, A5749, ABclonal Technology, China) and protein (A + G) agarose beads were added for immunoprecipitation. The agarose beads were removed and the precipitated proteins were mixed with SDS-PAGE buffer. The enriched proteins were eluted and denatured in a boiling water bath for 10 min to conduct immunoblotting.

The cell samples were collected by radio immunoprecipitation assay lysis buffer, containing protease and phosphatase inhibitors. Proteins were separated by SDS-PAGE and transferred to a 0.22 μm PVDF membrane. The PVDF membranes were then blocked with 2% BSA or 5% skim milk for 1 h. Antibodies were diluted and incubated overnight, membranes were washed and then incubated with secondary antibodies and visualized. The main antibodies used in this experiment included NF-κB pathway antibody sampler kit (9936, Cell Signaling Technology, USA) and rat anti-CVB5 VP1 (preserved in our laboratory).

### Immunofluorescence assay

The transfected cell samples were washed with Phosphate Buffered Saline (PBS), fixed with 4% paraformaldehyde for 30 min and 0.2% Triton X-100 for 20 min followed by blocking with 5% BSA for 2 h. Cells were washed with PBS, incubated with antibodies for 1 h, and washed with PBS containing 0.1% Tween-20 (PBS-T). Then cells were incubated with the secondary antibody (A23410 and A23220, Abbkine Scientific, China) for 1 h followed by washing with PBS-T. The nucleus was stained with diamidino-2-phenylindole (DAPI) for 2 min. Finally, the staining was observed with the Nikon confocal microscope.

### Real-time quantitative PCR (RT-qPCR)

RNAiso Plus reagent (9109, Takara, Japan) was used to extract total cellular RNA. Hifair III 1st Strand cDNA Synthesis SuperMix (11141ES10, Yeasen, China) was used for reverse transcription into cDNA. qPCR was performed in a mixture containing 2 μL forward primer, 2 μL reverse primer, 1 μL template cDNA and 5 μL SYBR Green Master Mix, following 95℃ 30 s; 95℃ 10 s, 60℃ 34 s (40 cycles). The data were normalized to the expression of the housekeeping gene *GAPDH*. The primer sequences are shown in Table 1.

**Table 1:** Primers used in this study

Genes	Primer sequence (5′–3′)
GAPDH	F: GAGTCAACGGATTTGGTCGT R: GACAAGCTTCCCGTTCTCAG
CVB5 VP1	F: CCAGTGCCCACGAAATAAA R: TTGCCTATGCTGATGAACGGT
EV71 VP1	F: GCAGCGGAACCGACTACTTTG R: GCCTGYCTAAGRCCTGCGAA
TAB1	F: AAAGCCCGACCTTAACCCTG R: GCTCCAGGCGGTAAAACT
TNF-α	F: GCCACCACGCTCTTCTGTCTAC R: GGGTCTGGGCCATAGAACTGAT
IL-1β	F: ACCTTCCAGGATGAGGACATGA R: CTAATGGGAACGTCACACACCA
IL-6	F: CACATGTTCTCTGGGAAATCG R: TTGTATCTCTGGAAGTTTCAGATTGTT
CCL5	F: CGCTGTCATCCTCATTGCTA R: CCATTTCTTCTCTGGGTTGG
TAB1-ALA	F: GTCCGCAGAGCTCCTGGCGGCCCAGCTGGCTGCCGCGCACGCCGAGGCCGATGTG R: CACATCGGCCTCGGCGTGCGCGGCAGCCAGCTGGGCCGCCAGGAGCTCTGCGGAC
OASL	F: TTGTGCCTGCCTACAGAGC R: TTCAGCTTAGTTGGCCGATGT
MXA	F: TTCAGCACCTGATGGCCTATC R: TGGATGATCAAAGGGATGTGG
ISG15	F: CTCTGAGCATCCTGGTGAGGAA R: AAGGTCAGCCAGAACAGGTCGT
ISG20	F: TGACCTGAAGCACGACTTCC R: CAGGCTGTTCTGGATGCTCT
IFIT1	F: TCTCAGAGGAGCCTGGCTAAG R: CCACACTGTATTTGGTGTCTAGG
IFIT2	F: ACCTCTGGACTGGCAATAGC R: GTCAGGATTCAGCCGAATGG
IFITM3	F: CATCGTCATCCCAGTGCTGAT R: ATGGAAGTTGGAGTACGTGGG

### Enzyme linked immunosorbent assay (ELISA)

To quantify pro-inflammatory factors, cells were transfected with plasmids, and culture supernatants were collected. The pro-inflammatory factors production was measured by Human TNF-α (MM-0122H1), IL-1β (MM-0181H1), IL-6 (MM-0049H1), and CCL5 (MM-14376H1) ELISA kit (MEIMIAN, China) in accordance with the manufacturer's instructions.

### Statistical analysis

All experiments were carried out three independent experiments and data were presented as mean ± SD. The Student’s t-test was performed for statistical analysis using the GraphPad Prism9 software. A *p*-values ≤ 0.05 and 0.01 was considered statistically significant and marked as * and **.

## Results

### Identification of proteins interacting with CVB5 3D polymerase

**Fig. 1:**
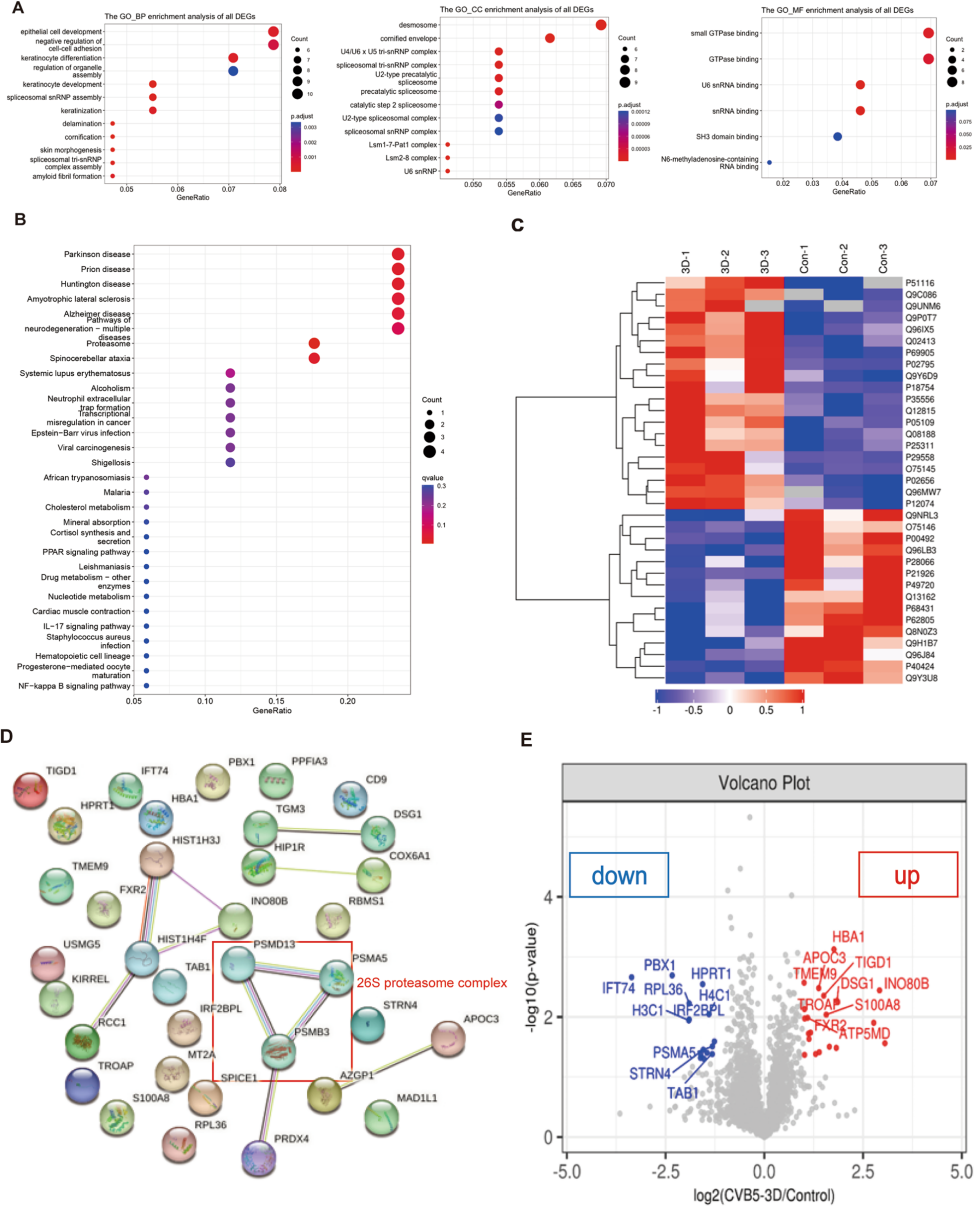
Identification of cellular proteins interacting with CVB5 3D polymerase. **A** GO functional enrichment analysis of the 116 differentially expressed proteins (BP, CC, and MF). A lower p-value indicates higher data reliability; **B** KEGG enrichment analysis of the 116 differentially expressed proteins. A lower p-value indicates higher data reliability; **C** The 35 significantly differentially expressed proteins clustering analysis between the CVB5 3D polymerase groups and controls (the Fold Change > 2.0 and p-value < 0.05). The red or blue colors represent significantly up-regulated or down-regulated proteins, respectively; **D** Protein–protein interaction (PPI) network of the 35 differentially expressed proteins. Green, red, and blue represent gene neighborhoods, gene fusions, and gene co-occurrence, respectively; **E** Volcano plots of the top 10 up or down-regulated proteins. Red dots for the significantly up-regulated proteins, Blue dots for the significantly down-regulated proteins, Gray dots for the nondifferential expressed proteins

We performed a proteomics analysis to identify host proteins that interact with CVB5 3D polymerase. Firstly, the CV5 3D polymerase was successfully overexpressed in RD cells (Additional file 1: Figure S1) and then followed the procedure of Additional file 2: Figure S2 to definite the differentially expressed proteins and bioinformatics analyses. From the Venn diagram showed 3296 proteins were commonly expressed between the CVB5 3D polymerase groups and the control groups, and 116 proteins were identified as differentially expressed (Additional file 3: Figure S3). These 116 differentially expressed proteins categorized under BP were mostly involved in cell development and cell adhesion, distributed in desmosome and envelope for CC, and participated in GTPase binding for MF (Fig. 1A). Also, the KEGG pathway enrichment analysis showed that the proteins were largely involved in nerve diseases, such as Parkinson disease, prion disease, Huntington disease and neurodegeneration-multiple diseases (Fig. 1B).

Among the 116 differentially expressed proteins, cluster analysis plots showed that a total of 35 proteins were significantly differentially expressed according to the Fold Change > 2.0 and *p*-value < 0.05. Of these proteins, 20 proteins were up-regulated and 15 proteins were down-regulated (Fig. 1C). As protein–protein interactions can result in the formation of complexes that exert biological regulation, we further used PPI networks to illustrate the cellular proteins interacting with 3D polymerase. As shown in Fig. 1D, the proteins involved in metabolic processes and immune regulation, such as the components of the 26S proteasome complex, have multiple protein hydrolase activities and are dependent on the ubiquitin pathway. Then, we selected the top 10 significantly down-regulated or up-regulated proteins (Table 2) for analysis and visualization with a Volcano plot (Fig. 1E). Based on functional analysis, we identified a candidate protein, TAB1, as a cellular protein interacting with CVB5 3D polymerase.

**Table 2:** The top 10 down-regulated or up-regulated proteins

Gene name	Protein name	Protein	*p*-values	Level
PBX1	Pre-B-cell leukemia transcription factor 1	Q96LB3	0.0020	Down
IFT74	Infraglabellar transport protein 74 homolog	Q96LB3	0.0022	Down
HPRT1	Hypoxanthine–guanine phosphoribosyl transferase	P00492	0.0028	Down
RPL36	60S ribosomal protein L36	Q9Y3U8	0.0059	Down
H4C1	Histone H4	P62805	0.0090	Down
IRF2BPL	Probable E3 ubiquitin-protein ligase IRF2BPL	Q9H1B7	0.0110	Down
H3C1	Histone H3.1	P68431	0.0114	Down
PSMA5	Proteasome subunit alpha type-5	P28066	0.0309	Down
STRN4	Striatin-4	Q9NRL3	0.0352	Down
TAB1	TGF-beta-activated kinase 1 and MAP3K7-binding protein 1	Q15750	0.0360	Down
HBA1	Hemoglobin subunit alpha	P69905	0.0007	Up
TMEM9	Proton-transporting V-type ATPase complex assembly regulator TMEM9	Q9P0T7	0.0027	Up
APOC3	Apolipoprotein C-III	P02656	0.0033	Up
INO80B	INO80 complex subunit B	Q9C086	0.0036	Up
TIGD1	Tigger transposable element-derived protein 1	Q96MW7	0.0044	Up
DSG1	Desmoglein-1	Q02413	0.0054	Up
TROAP	Tastin	Q12815	0.0074	Up
S100A8	Protein S100-A8	P05109	0.0091	Up
ATP5MD	ATP synthase membrane subunit DAPIT, mitochondrial	Q96IX5	0.0103	Up
FXR2	Fragile X mental retardation syndrome-related protein 2	P51116	0.0106	Up

### CVB5 3D polymerase down-regulates TAB1 expression through the lysosomal pathway

**Fig. 2:**
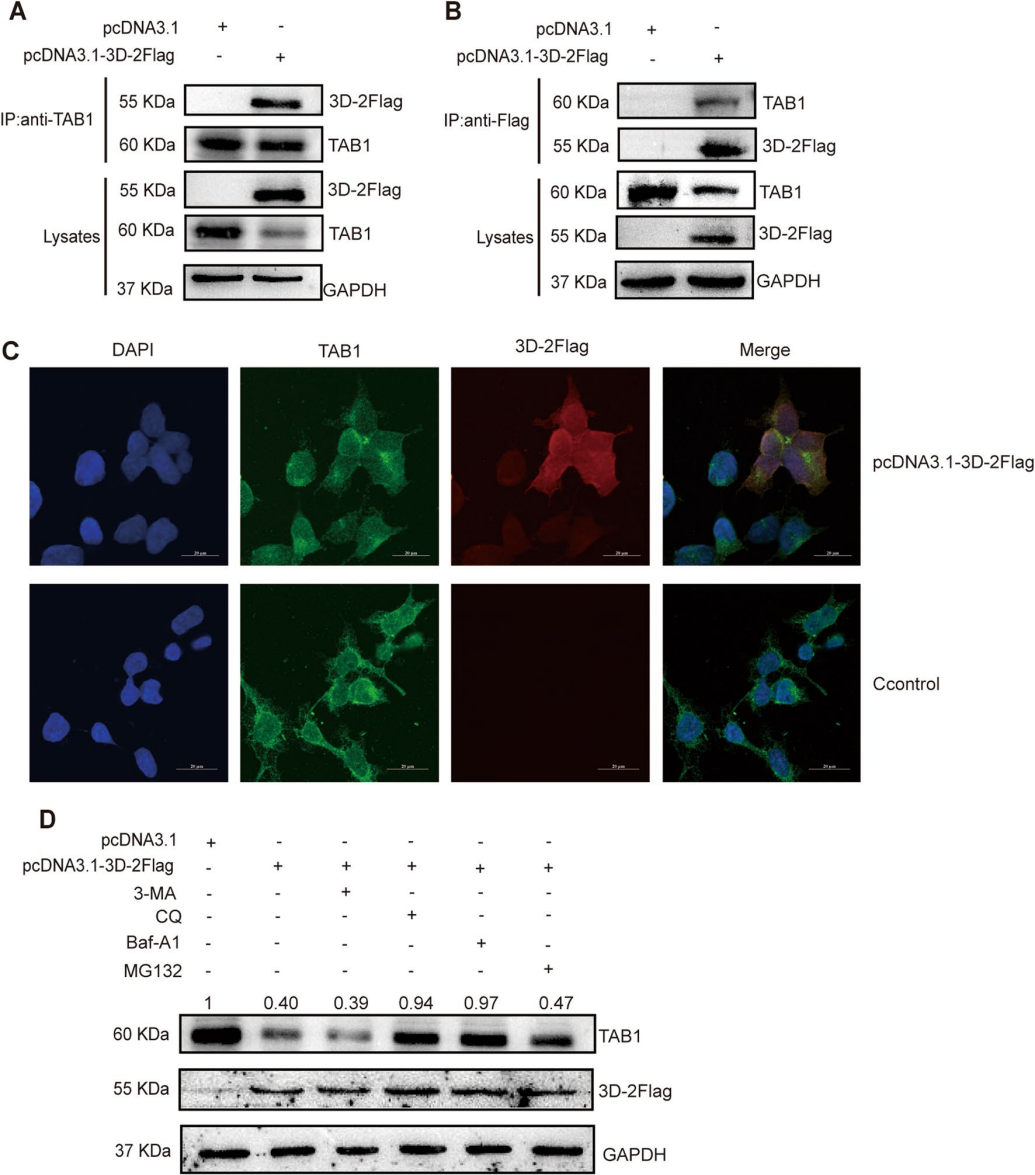
CVB5 3D polymerase down-regulates TAB1 expression through the lysosomal pathway. pcDNA3.1-3D-2Flag (pcDNA3.1 as the control) was transfected into RD cells and harvested at 24 h post-transfection. **A** Lysates were subjected to anti-TAB1 immunoprecipitation and analyzed by immunoblotting; **B** Lysates were subjected to anti-Flag immunoprecipitation and analyzed by immunoblotting; **C** The co-localization of 3D polymerase and TAB1 analyzed by confocal assay; **D** Cells maintained for 24 h in the presence of the autophagy inhibitor (3MA), lysosomal inhibitor (CQ or Baf-A1), and protease inhibitor (MG132). The expression of TAB1 in cell lysates was analyzed by Western blotting. The numbers are the level of expression compare to the controls

**Fig. 3:**
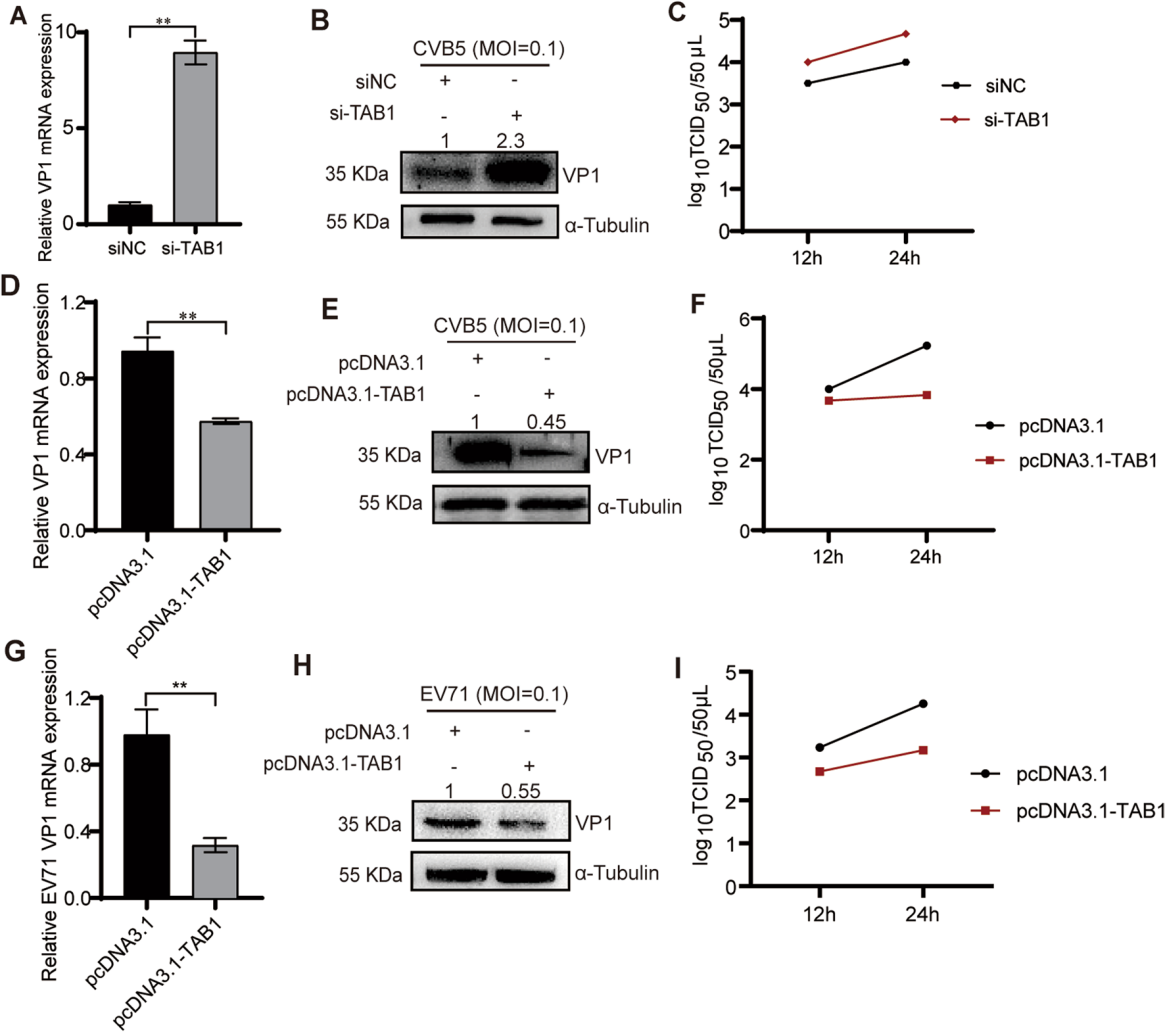
TAB1 inhibits CVB5 replication. **A**–**C** si-TAB1 (siNC as the control) was transfected into RD cells, then infected CVB5. Cells were harvested at 24 h.p.i.. The expression of CVB5 VP1 in cell lysates was analyzed by RT-qPCR (**A**) and Western blotting (**B**); Supernatant was collected for TCID50 measurement (**C**); (**D**–**F**) pcDNA3.1-TAB1 plasmid (pcDNA3.1 as the control) was transfected into RD cells, then infected CVB5. Cells were harvested at 24 h.p.i.. The expression of CVB5 VP1 in cell lysates was analyzed by RT-qPCR (**D**) and Western blotting (**E**); Supernatant was collected for TCID50 measurement (**F**); **G**–**I** pcDNA3.1-TAB1 (pcDNA3.1 as the control) was transfected into RD cells, then infected EV71 (MOI = 0.1). Cells were harvested at 24 h.p.i.. The expression of EV71 VP1 in cell lysates was analyzed by RT-qPCR (**G**) and Western blotting (**H**); Supernatant was collected for TCID50 measurement (**I**). Data are represented as mean ± SD. **P ≤ 0.01

To confirm the interaction between TAB1 and 3D polymerase, we overexpressed the pcDNA3.1-3D-2Flag and conducted co-immunoprecipitation to validate the associations. As shown in Figs. 2A, 3D polymerase directly specifically interacted with TAB1 which was detected by using the anti- TAB1 antibody. To further confirm the TAB1 direct association with 3D polymerase, the anti-Flag was used to perform co-immunoprecipitation, and results confirmed that TAB1 was also specifically bound to 3D polymerase (Fig. 2B). Also, we examined their co-localization by confocal assay and observed that TAB1 was mainly distributed in the cytoplasm and 3D polymerase facilitated the TAB1 entry into the nucleus (Fig. 2C).

From the cluster analysis plots (Fig. 1E), the 3D polymerase down-regulated the expression of TAB1. Consistent with the plots, the 3D polymerase down-regulated TAB1 at the protein levels (Fig. 2D), not at the RNA level (Additional file 4: Figure S4). To determine which pathways contributed to the down-regulation of TAB1, the autophagy inhibitor (3MA), lysosomal inhibitors (CQ and Baf-A1), and the protease inhibitor (MG132) were evaluated. The lysosomal inhibitors (CQ and Baf-A1) restored the expression of TAB1 from the protein level, proposing that 3D polymerase down-regulated TAB1 expression through the lysosomal pathway (Fig. 3D and Additional file 5: Figure S5).

### TAB1 inhibits CVB5 replication

To evaluate the effect of TAB1 on viral replication, endogenous knockdown (si-TAB1) or overexpression of TAB1 (pcDNA3.1-TAB1) was successfully performed, and si-TAB1-3 was selected for experiments (Additional files 6, 7: Figure S6 and S7). As shown in Fig. 3A–B, after transfection with si- TAB1, the mRNA and protein expressions of CVB5 VP1 promoted significantly. Also, the TCID_50_ showed that the viral titers increased after 12 and 24 h compared to the control (Fig. 3C). Meanwhile, after transfection with pcDNA3.1-TAB1, the mRNA and protein expressions of CVB5 VP1 were inhibited significantly (Fig. 3D–E). And, the TCID_50_ showed that the viral titers decreased after 12 and 24 h compared to the control (Fig. 3F). Also, TAB1 inhibited CVB5 cytopathic effect (CPE) (Additional file 8: Figure S8). These results show that TAB1 inhibits the CVB5 replication. Furthermore, we investigated whether TAB1 regulated EV71 replication. As shown in Fig. 3G–H, TAB1 could inhibit mRNA expression as well as protein expression of EV71 VP1. Also, the TCID_50_ showed that TAB1 significantly decreased EV71 viral titers after 12 and 24 h compared to the control (Fig. 3I). All results indicate that TAB1 may play a vital role in HFMD viral replication.

### TAB1 promotes the expression of pro-inflammatory factors and activates the NF-κB pathway

**Fig. 4:**
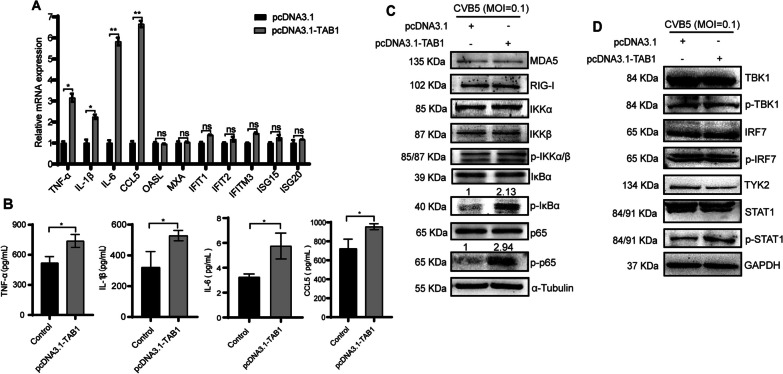
TAB1 promotes the expressions of pro-inflammatory factors and activates the NF-κB pathway. pcDNA3.1-TAB1 (pcDNA3.1 as the control) was transfected into RD cells, then infected CVB5. Cells were harvested at 24 h.p.i.. **A** The expressions of pro-inflammatory factors and ISGs were analyzed by RT-qPCR; **B** ELISA analysis of the production of TNF-α, IL-1β, IL-6, and CCL5 in the supernatant; **C** The expressions of NF-κB pathway proteins in cell lysates were analyzed by Western blotting; **D** The expressions of IFN-I pathway proteins in cell lysates were analyzed by Western blotting. Data are represented as mean ± SD. *P ≤ 0.05, **P ≤ 0.01

Interferon-stimulated genes (ISGs) and pro-inflammatory factors are important regulators to inhibit viral infection. We examined these factors and showed that the expressions of inflammatory factors were up-regulated, especially the late-stage pro-inflammatory factors (IL-6 and CCL5) (Fig. 4A). Additionally, the ELISA results confirmed TAB1 induced the production of TNF-α, IL-1β, IL-6, and CCL5 (Fig. 4B). These results suggested that TAB1 might regulate the NF-κB pathway to promote the expression of pro-inflammatory factors. This was confirmed by the observation that TAB1 stimulated the phosphorylation of IκBα, which was then degraded by the proteasome, thereby activating the phosphorylation of p65, which activated the expression of downstream inflammatory factors that exerted antiviral effects (Fig. 4C). Concurrently, we observed that TAB1 did not affect the expression of the key proteins in the IFN-I pathway (Fig. 4D). All results showed that TAB1 promoted the expression of pro-inflammatory factors and activates the NF-κB pathway.

### The 90-96aa domain of TAB1 is responsible for the function

**Fig. 5:**
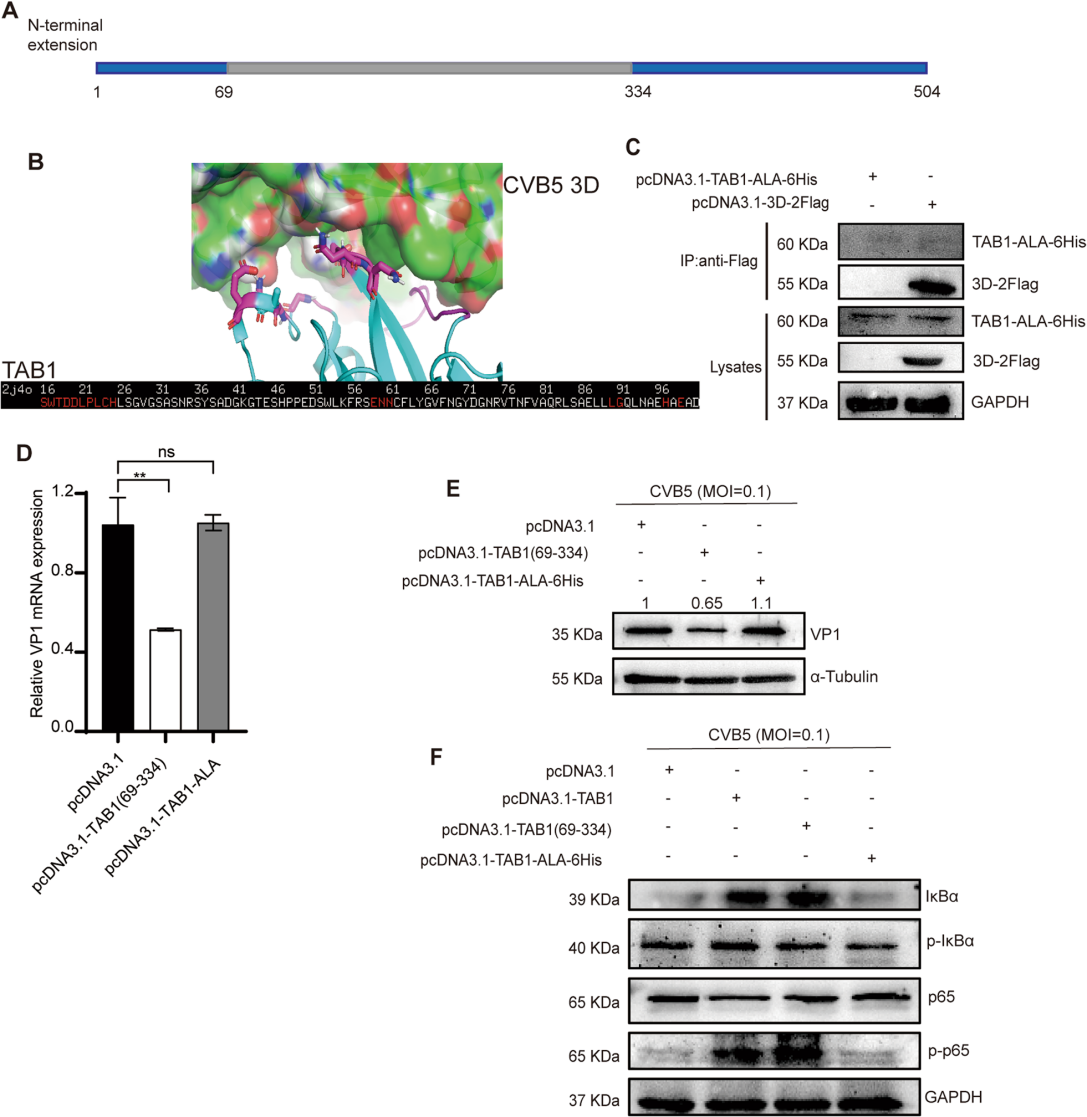
The 90-96aa domain of TAB1 is responsible for the function. **A** The diagram of TAB1 protein structure; **B** Interaction between TAB1 and 3D polymerase as predicted by Pymol; **C** pcDNA3.1-3D-2Flag or pcDNA3.1- TAB1-ALA-6His (pcDNA3.1 as the control) was transfected into RD cells. Cells were harvested at 24 h post-transfection and lysed. Lysates were subjected to anti-Flag immunoprecipitation and analyzed by immunoblotting; **D**–**E** pcDNA3.1- TAB1(69–334) or pcDNA3.1- TAB1- ALA (pcDNA3.1 as the control) was transfected into RD cells, then infected CVB5. Cells were harvested at 24 h.p.i.. The expression of CVB5 VP1 in cell lysates was analyzed by RT-qPCR (**D**) and Western blotting (**E**); **F** pcDNA3.1- TAB1, pcDNA3.1- TAB1(69–334), or pcDNA3.1- TAB1-ALA-6His (pcDNA3.1 as the control) was transfected into RD cells, and then infected CVB5. Cells were harvested at 24 h.p.i.. The expressions of NF-κB pathway proteins in cell lysates were analyzed by Western blotting. Data are represented as mean ± SD. **P ≤ 0.01

We performed structural domain delineation of TAB1 based on the domain mapping of the disease mutations database. TAB1 is composed of 504 amino acid (aa) residues and contains the phosphatase 2C (PP2C) domain which has phosphatase activity (Fig. 5A). Meanwhile, Pymol was used to predict the interaction domains of 3D polymerase and TAB1. The results indicated that the 16-25aa domain (SWTDDLPLCH), 59-61aa domain (ENN), and 90-96aa domain (LGQLNAEHAL) of TAB1 might enable the interaction between the two proteins (Fig. 5B). The 3D polymerase is an RNA synthesis polymerase, so we speculated that the 90-96aa domain located in the active structural domain of TAB1 phosphatase might be their interaction domain. Therefore, the alanine residue within the 90-96aa domain of TAB1 was mutated and constructed with His tag into pcDNA3.1 (pcDNA3.1- TAB1-ALA-6His). The co-immunoprecipitation results showed that the mutation of the 90-96aa of TAB1 and 3D polymerase could not be immunoprecipitated (Fig. 5C) which agreed with our prediction. All results illustrated that CVB5 3D polymerase mainly interacted with the 90-96aa domain of TAB1.

Also, we tested the effect of the 90-96aa and 90-96aa-ALA mutant of TAB1 on CVB5 replication. The results showed that mutating the alanine residue within the 90-96aa domain restricted TAB1 from inhibiting viral replication (Fig. 5D–E), further suggesting that the 90-96aa domain is vital for TAB1 function. Moreover, the TAB1 90-96aa mutant did not activate the NF-κB pathway through the phosphorylation of IκBα and p65 (Fig. 5F). These results confirmed the function of the TAB1 90-96aa domain.

## Discussion

CVB5 infection is a serious threat to global public health, especially in infants and young children with HFMD, resulting in neurological complications. However, the mechanisms of CVB5 in causing HFMD and even severe diseases have not been fully explored. Accumulated evidence implied the manipulation of viral replication processes by the host chiefly occurs through physical interactions between the viral and host proteins. Among the several NSPs of CVB5, the 3D polymerase is a virus-encoded RNA-dependent RNA polymerase (RdRP) which plays an important role in the lifecycle of picornavirus involved in viral structural integrity, nucleotide recognition and binding, phosphoryl transfer, and initiation of nucleotide binding function [23]. Crystal structure analysis revealed that the structure of 3D polymerase resembled a "right hand" structure consisting of "palm", "finger" and "thumb" domains, which determined the correct geometric arrangement of substrate molecules and metal ions at the active catalytic site [24]. Previous studies have revealed that the picornavirus 3D polymerase could interact with host proteins to patriciate with viral RNA synthesis. For example, 3D polymerase promotes EV71 infection by interacting with the host protein ANXA2 to assist in the assembly of replicative organelles [25]. Also, 3D polymerase plays a role in regulating IFN-I signaling pathways as well as viral replication. For example, EV71 3D polymerase interacts with MDA5 to inhibit IFN-β promoter activation and mRNA expression [26]. Simultaneously, the EV-D68 3D polymerase inhibited the expression of IFN-β by affecting the expression of PGAM5 [27]. Collectively, the picornavirus 3D polymerase might broadly associate with host factors to regulate viral replication. Therefore, we focused on understanding the interaction between 3D polymerase of CVB5 with host factors involved in the innate immune pathway.

Recently LC–MS/MS protein quantification technology has contributed to a number of studies on protein–protein interactions [28]. Several studies have reported on the use of LC–MS to identify and characterize host and viral proteins. Such as LC–MS/MS quantitative proteomics has been used to reveal changes in the nucleolar proteome of influenza A virus, hepatitis B virus, and human immunodeficiency virus-infected cells [29–31]. Hence, we used LC–MS/MS proteomics technology to screen for host proteins that interact with CVB5 3D polymerase in RD cells. A total of 116 proteins with different expressions were identified, of which 35 proteins exhibited significant expression differences. We enriched the immune pathway for IMPDH2 (Inosine-5′-monophosphate dehydrogenase 2), ATG5 (Autophagy Related 5), TAB1, and other proteins. IMPDH2 is an important enzyme in guanine nucleotide biosynthesis and up-regulated in a variety of tumor cells [32–34]. It promotes the development of colorectal cancer through activation of PI3K/AKT signaling pathways [35]. Recent studies have also shown that IMPDH2 regulates innate immune signaling pathways and promotes the replication of the SARS-CoV-2 virus [36]. ATG5 is a key autophagic factor that regulates dendritic cell reprogramming to inhibit respiratory syncytial virus infection [37]. Collectively, the identified cellular proteins and how they interact with CVB5 3D polymerase may be an important strategy to explore the mechanism of viral replication and guide antiviral drug development in the further.

TAB1 is constitutively associated with the composition N-terminal kinase domain of TAK1 and can be activated by pro-inflammatory cytokines (TNFα and IL-1β) and toll-like receptor ligands. The TAB1-TAK1 complexes play essential roles in the activation of inflammatory responses and involve in the regulation of a wide range of pathological processes, such as cancer and diabetes [38–40]. Furthermore, The NSP5 proteases of SARS-CoV-2 could mediate the cleavage of TAB1 which point to enhance production of cytokines and inflammatory response in COVID-19 patients, demonstrating that they involved in the host innate immune response [41]. And, TAB1-mediated p38α activation facilitated Hepatitis C Virus replication [42]. However, its regulatory mechanism in antiviral immunity via other inflammatory responses has not been reported previously. Our study demonstrated that TAB1 down-regulated by CVB5 3D polymerase stimulated the phosphorylation of IκBα, followed by activation of the NF-κB pathway and proinflammatory factors. Thus, TAB1 is a key cellular protein that bridges the lifecycle of the HFMD virus and the host innate immune response.

## Conclusion

**Fig. 6:**
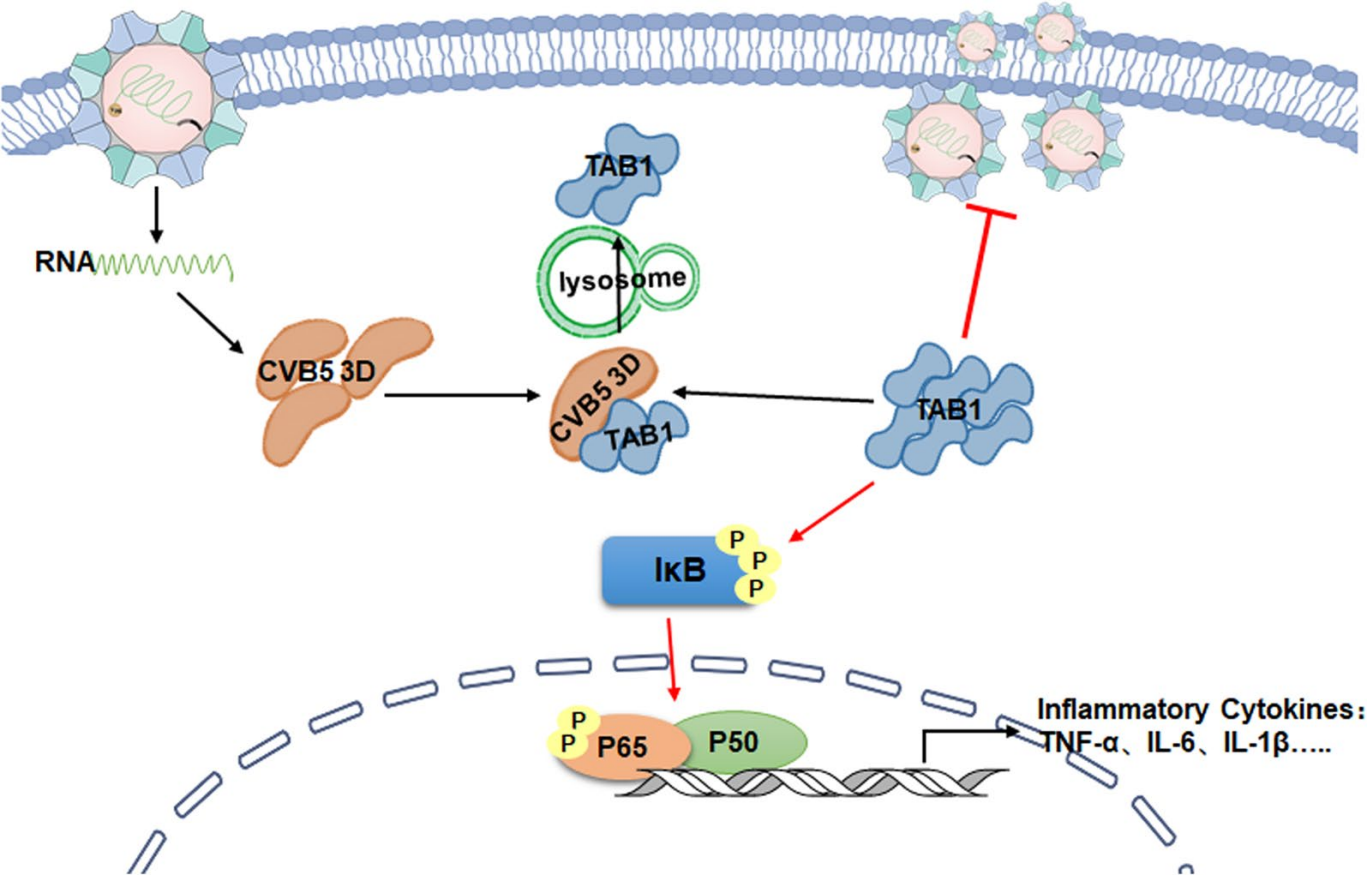
A proposed model for the TAB1 inhibition CVB5 replication. CVB5 3D polymerase interacts with TAB1 and down-regulates TAB1 expression in cells. TAB1 stimulated the phosphorylation of IκBα, thereby activating the phosphorylation of p65, which activated the expression of downstream inflammatory factors that exerted antiviral effects

In summary, we investigated the cellular protein TAB1 was one of the down-regulated proteins associated with CVB5 3D polymerase in RD cells. TAB1 stimulated the phosphorylation of IκBα, followed by activation of the NF-κB pathway and proinflammatory factors, then inhibited HFMD viral replication including EV71 and CVB5 (Fig. 6). Further experimental data showed that the 90-96aa domain of TAB1 plays a vital role. Our study provides new insight into the interaction network of virus and host to enable HFMD drug development in the future.

### Supplementary Information

**Additional file 1**. **Figure S1**: pcDNA3.1-3D-2Flag was transfected into RD cells and harvested at 24hours post-transfection (pcDNA3.1 as the control). The expression of 3D-2Flag was analyzed by Western blotting.

**Additional file 2**. **Figure S2:** Flowchart of the bioinformatic analysis to define the differentially expressed proteins.

**Additional file 3**. **Figure S3**: Venn diagrams showed the numbers of overlapped proteins between the CVB5 3D groups and controls.

**Additional file 4**. **Figure S4:** pcDNA3.1-3D-2Flag was transfected into RD cells and harvested at 24hours post-transfection (pcDNA3.1 as the control). The expression of TAB1 was analyzed by RT-qPCR.

**Additional file 5**. **Figure S5**: pcDNA3.1-3D-2Flag (pcDNA3.1 as the control) was transfected into RD cells and harvested at 24hours post-transfection. The expression of TAB1, LC3II/I, p62 and LAMP2 were analyzed by Western blotting.

**Additional file 6**. **Figure S6:** si-TAB1 (siNC as the control) was transfected into RD cells and harvested at 24hours post-transfection. The expression of TAB1 was analyzed by RT-qPCR (A) and Western blotting (B). Data are represented as mean ± SD. **P ≤ 0.01.

**Additional file 7**. **Figure S7:** pcDNA3.1-TAB1 (pcDNA3.1 as the control) was transfected into RD cells and harvested at 24hours post-transfection. The expression of TAB1 was analyzed by RT-qPCR (A) and Western blotting (B). Data are represented as mean ± SD. ***P ≤ 0.001.

**Additional file 8**. **Figure S8**: pcDNA3.1-TAB1 (pcDNA3.1 as the control) was transfected into RD cells and the supernatant was collected for CPE experiments at 24 hours post-transfection. Data are represented as mean ± SD. *P ≤ 0.05.

## Data Availability

All data and materials were available from the corresponding author on reasonable request unless purchased from a commercial entity. The protein data presented in this study are available in the iProX under PXD044375.

## Abbreviations

CVB5: Coxsackievirus group B type 5
HFMD: Hand-foot-mouth disease
TAB1: TGF-BATA-activated kinase1 binding protein 1
NF-κB: Nuclear factor kappa-B
NSPs: Nonstructural proteins
IFN-I: Type I interferon
LC–MS/MS: Liquid chromatography-tandem mass spectrometry
MOI: Multiplicity of infection
GO: Gene ontology
KEGG: Kyoto encyclopedia of genes and genomes
ISGs: Interferon-stimulated genes
