# Supplementary figures and images for: Down-regulated TAB1 suppresses the replication of Coxsackievirus B5 via activating the NF-κB pathways through interaction with viral 3D polymerase
Source: Virol J. 2023 Dec 10;20:291. doi: 10.1186/s12985-023-02259-w (PMC10712077; doi:10.1186/s12985-023-02259-w)

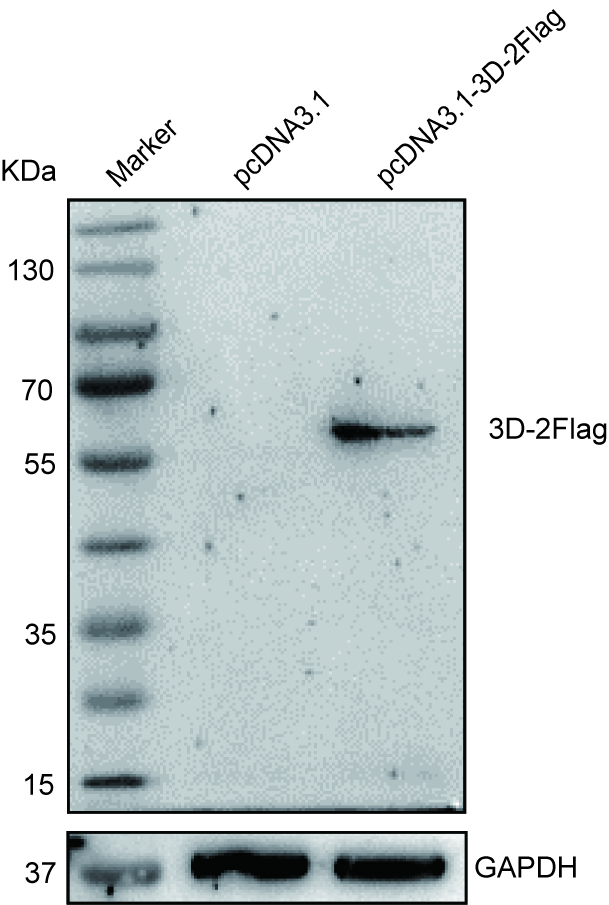

Supplement: Supplementary file 1 — Additional file 1. Figure S1: pcDNA3.1-3D-2Flag was transfected into RD cells and harvested at 24hours post-transfection (pcDNA3.1 as the control). The expression of 3D-2Flag was analyzed by Western blotting. [file 12985_2023_2259_MOESM1_ESM.tif]

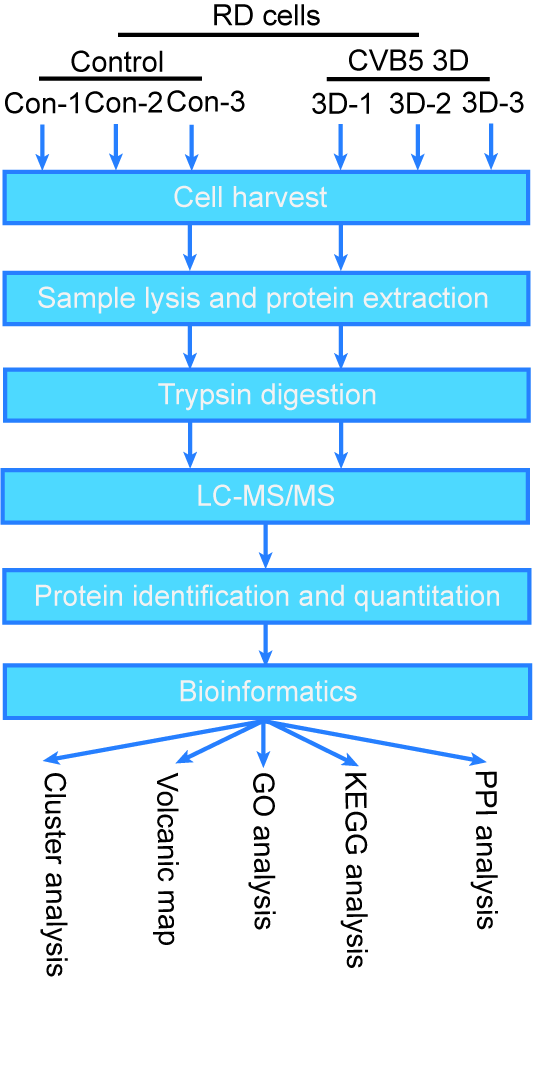

Supplement: Supplementary file 2 — Additional file 2. Figure S2: Flowchart of the bioinformatic analysis to define the differentially expressed proteins. [file 12985_2023_2259_MOESM2_ESM.tif]

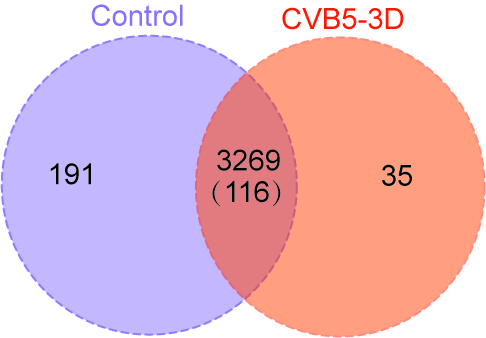

Supplement: Supplementary file 3 — Additional file 3. Figure S3: Venn diagrams showed the numbers of overlapped proteins between the CVB5 3D groups and controls. [file 12985_2023_2259_MOESM3_ESM.tif]

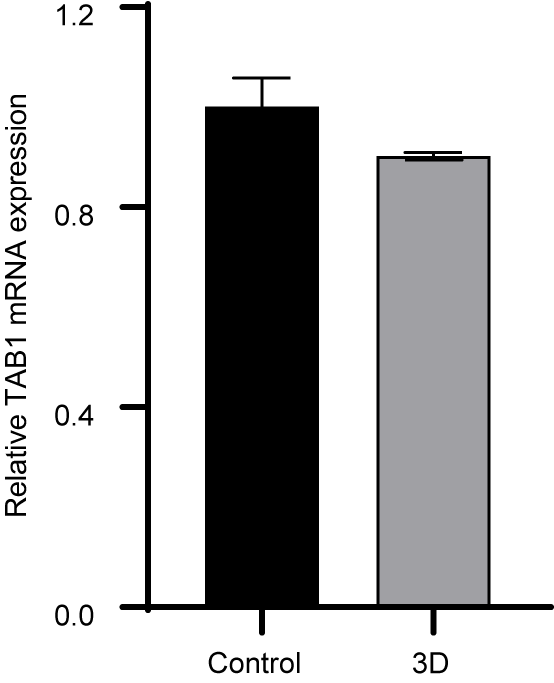

Supplement: Supplementary file 4 — Additional file 4. Figure S4: pcDNA3.1-3D-2Flag was transfected into RD cells and harvested at 24hours post-transfection (pcDNA3.1 as the control). The expression of TAB1 was analyzed by RT-qPCR. [file 12985_2023_2259_MOESM4_ESM.tif]

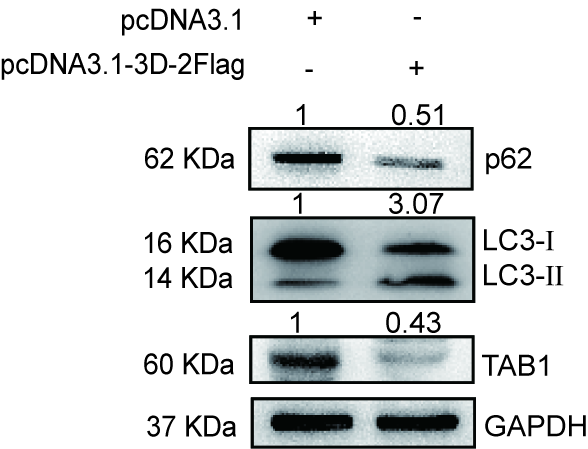

Supplement: Supplementary file 5 — Additional file 5. Figure S5: pcDNA3.1-3D-2Flag (pcDNA3.1 as the control) was transfected into RD cells and harvested at 24hours post-transfection. The expression of TAB1, LC3II/I, p62 and LAMP2 were analyzed by Western blotting. [file 12985_2023_2259_MOESM5_ESM.tif]

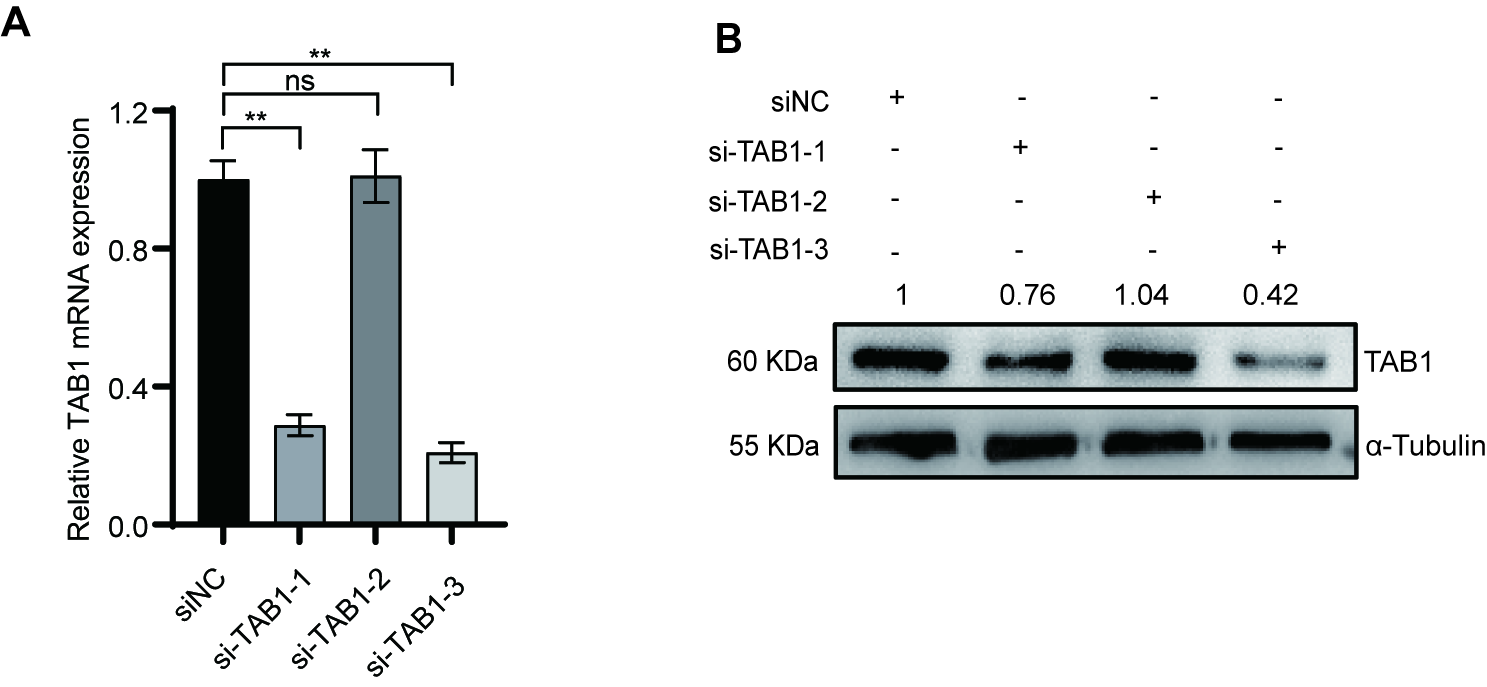

Supplement: Supplementary file 6 — Additional file 6. Figure S6: si-TAB1 (siNC as the control) was transfected into RD cells and harvested at 24hours post-transfection. The expression of TAB1 was analyzed by RT-qPCR (A) and Western blotting (B). Data are represented as mean ± SD. **P ≤ 0.01. [file 12985_2023_2259_MOESM6_ESM.tif]

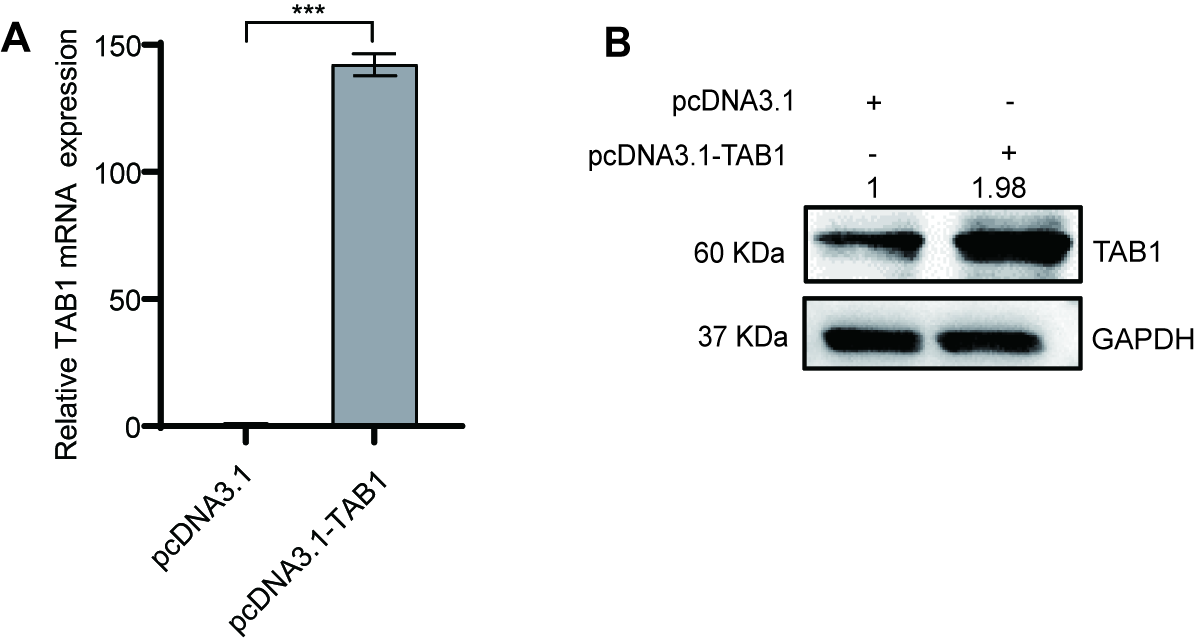

Supplement: Supplementary file 7 — Additional file 7. Figure S7: pcDNA3.1-TAB1 (pcDNA3.1 as the control) was transfected into RD cells and harvested at 24hours post-transfection. The expression of TAB1 was analyzed by RT-qPCR (A) and Western blotting (B). Data are represented as mean ± SD. ***P ≤ 0.001. [file 12985_2023_2259_MOESM7_ESM.tif]

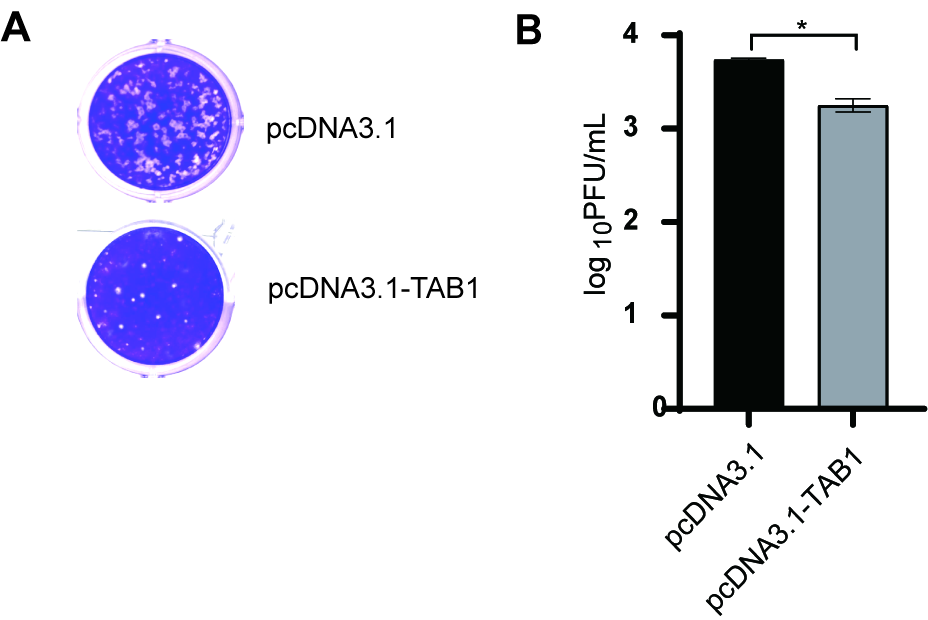

Supplement: Supplementary file 8 — Additional file 8. Figure S8: pcDNA3.1-TAB1 (pcDNA3.1 as the control) was transfected into RD cells and the supernatant was collected for CPE experiments at 24 hours post-transfection. Data are represented as mean ± SD. *P ≤ 0.05. [file 12985_2023_2259_MOESM8_ESM.tif]
